# Perinatal Palliative Care: Barriers and Attitudes of Neonatologists and Nurses in Poland

**DOI:** 10.1155/2013/168060

**Published:** 2013-10-28

**Authors:** Aleksandra Korzeniewska-Eksterowicz, Maria Respondek-Liberska, Łukasz Przysło, Wojciech Fendler, Wojciech Młynarski, Ewa Gulczyńska

**Affiliations:** ^1^Pediatric Palliative Care Unit, Department of Pediatrics, Oncology, Hematology and Diabetology, Medical University of Lodz, 36/50 Sporna Street, 91-738 Lodz, Poland; ^2^Gajusz Foundation, Pediatric Palliative Care Center, Home Hospice for Children of Lodz Region, 87 Dąbrowskiego Street, 93-271 Lodz, Poland; ^3^Department for Diagnosis and Prevention of Congenital Malformations, Medical University of Lodz and Research Institute of Polish Mother's Memorial Hospital, 281/289 Rzgowska Street, 93-338 Lodz, Poland; ^4^Department of Pediatrics, Oncology, Hematology and Diabetology, Medical University of Lodz, 36/50 Sporna Street, 91-738 Lodz, Poland; ^5^Department of Neonatology, Research Institute of Polish Mother's Memorial Hospital, 281/289 Rzgowska Street, 93-338 Lodz, Poland

## Abstract

*Objective*. To identify barriers and personnel attitudes towards realization of palliative care principles in neonatological units. *Study Design*. An anonymous questionnaire was posted to all heads of departments and head nurses of all the 27 neonatological units in the Lodz area. *Results*. We received 46 (85%) questionnaires. Final analysis comprised 42 properly filled-in questionnaires (by 22 doctors and 20 nurses). In case of prenatal diagnosis of a lethal defect, 77.27% of doctors and 65% of nurses opted for informing the mother also about the possibility of pregnancy continuation and organization of palliative care after delivery. Most of respondents accepted conditions for abortion pointed by the Polish law. The most common barriers pointed out by both groups were insufficient knowledge of the personnel on palliative medicine and family preference for life sustaining treatment. *Conclusions*. Understanding attitudes of personnel towards palliative care and identification of barriers are a starting point for future efforts to improve the system of neonatological care.

## 1. Introduction

Recent advances in prenatal diagnostics and neonatal medicine have resulted in the formation of a new area of interest within pediatric palliative care, that is, perinatal palliative care (PPC) [1, 2]. The idea of PPC is not new and was developed in the 1980s. In 1982 Whitfield et al. [3] suggested “transfer” of adult palliative care concept into neonatal care; later in the United Sates the movement of perinatal hospices developed [4–8]. The last decade has witnessed increasing interest of authors in PPC, and numerous publications on this subject have appeared. In Poland palliative care for children has been mainly provided by specialized pediatric palliative home care teams and home-based model of care is called, “home hospice” [9]. The availability of home PPC in Poland is relatively good [9]; however, implementation of palliative care principles in hospital conditions may be problematic. The literature abounds in results of studies on barriers to introduction of palliative care elements in pediatric hospital care, in particular in neonatological setting or neonatological intensive care units (NICU) [10–13]. There are no reports concerning this problem in the Polish literature.

The aim of this study was to identify barriers and personnel attitudes towards realization of palliative care principles in neonatological units in the Lodz region. 

## 2. Material and Methods

We conducted a questionnaire study; an anonymous questionnaire was posted together with an addressed envelope with stamps to all heads of departments and head nurses of all the 27 neonatological units in the Lodz area. According to the concept of regionalized perinatal care, among those units, there were 14 of 1st level reference, 9 of 2nd level, and 2 of 3rd level. There were also included 2 additional units: 1 NICU intended for neonates with congenital malformation and 1 pediatric ICU.

Anonymous questionnaire was constructed for the needs of this research. The procedure of drawing the questionnaire up was begun from preparing a set of questions letting us make assumed analysis. Questions were prepared in the support about the literature review of applying perinatal palliative care, review of validated tools and clinical experience. Next a preliminary set of questions was presented for team of experts which showed questions most accurately referring to aims of the research. Prepared preliminary version of questionnaire was subjected to the pilot study under the executioner of understanding questions by 20 doctors and nurses. Respondents additionally assessed the graphical side and the easiness and the time of filling in a questionnaire. The questionnaire covered basic social and demographic data of the respondents and 22 closed questions on attitudes towards PPC and barriers to PPC implementation in a given ward. Social and demographic data included the age, gender, profession (physician or nurse), and Religion (Catholic, Orthodox, Protestant, Islamic, atheist, and others). Questions on attitudes towards PPC (except for the question on acceptance of abortion) were single choice questions (closed questions). The questionnaire listed 16 identified potential barriers to realization of PPC principles in neonatological wards and respondents were asked to determine their occurrence. The answers were categorized as (1) almost always, (2) frequently, (3) occasionally, and (4) never happening.

## 3. Statistical Analysis

Data was presented with mean and standard deviation or with number and percentage of observations. Two-tailed Fisher's exact test was used for comparisons on categorical variables. *P* values <0.05 were considered as statistically significant. All descriptive analyses were performed using STATISTICA for Windows release 6.0 software.

The study was approved by the ethics committee of Medical University of Lodz (RNN/136/10/KE).

## 4. Results

We sent a total of 54 questionnaires and had 46 (85%) returned. Final analysis comprised 42 properly filled-in questionnaires (by 22 doctors and 20 nurses). Social and demographic structures of the respondents have been presented in Table 1.

The respondents were asked for their cooperation with pediatric palliative home care teams. 95.45% of doctors and 80% of nurses had heard about these teams before, 72.73% of doctors and 80% of nurses declared earlier cooperation with the team, and it was the team from HHChLR. One-third of respondents did participate in training programmes within PPC. 

The respondents were asked about management in case of prenatal diagnosis of a lethal defect understood as (1) a developmental anomaly that leads to spontaneous abortion, immature delivery, or intrauterine death and (2) a developmental anomaly leading to the premature death of a live-born child, irrespective of any therapy that may be applied. If the defect was detected early, 77.27% of doctors and 65% of nurses opted for informing the mother also about the possibility of pregnancy continuation and organization of home hospice care after delivery. If the defect was undoubtedly lethal, 36.36% of doctors suggested the department closest to mother's place of residence as the optimal place of delivery. Nurses (45%) more frequently chose a multispecialist center. The vast majority of doctors (90.91%) and nurses (75%) claimed that diagnosis of a lethal defect is not an indication for a caesarean section. Respondents claimed that the best time for suggesting palliative care to the family in case of an unfavorable prenatal diagnosis is the time of pregnancy (68.18% of doctors and 75% of nurses). According to 45.45% of doctors and 40% of nurses, information about unfavorable prognosis and suggestion of palliative care should be communicated to the family by their physician together with the head of department and hospice care doctor. Qualification for palliative care according to 59.09% of doctors and 70% of nurses should be performed by a consulting team (the responsible physician, head of department, hospice doctor, and specialists depending on type of defect). Only in 45% of studied departments is there a possibility of including a psychologist, who in 60% of cases is employed in a given department and in 40% of cases works in the same hospital.

Respondents were asked to comment on specific aspects of PPC. Detailed results concerning opinions on withholding resuscitation are presented in Table 2.

**Figure 1:**
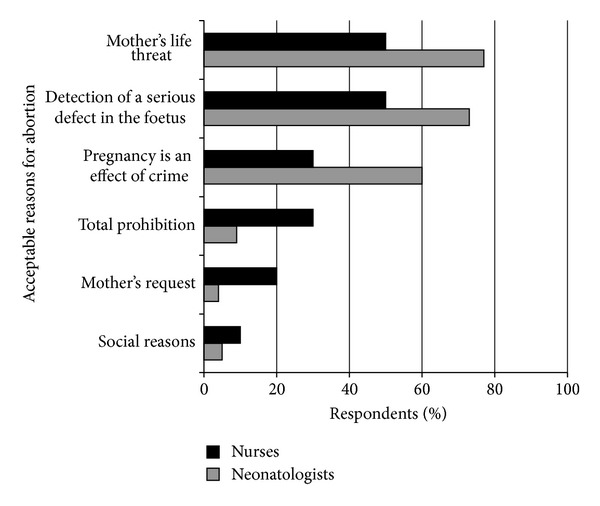
Reasons for abortion acceptable by respondents.

The respondents were asked to list conditions accepted by them when abortion should be possible and usually mentioned those accepted by the Polish law. The doctors were more liberal as compared with nurses; however, these differences were not statistically significant. The results have been presented in Figure 1. 

**Figure 2:**
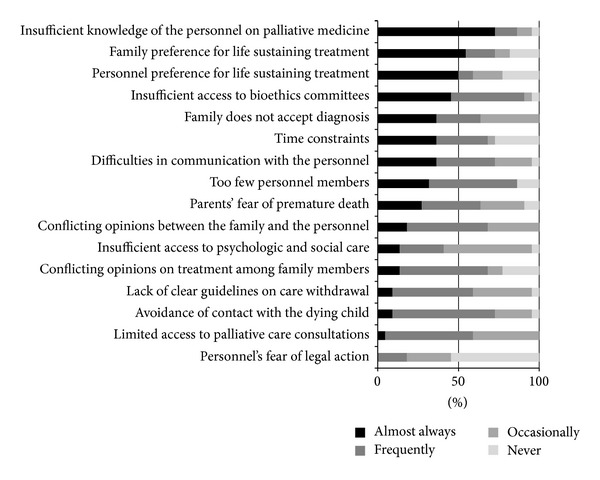
Barriers pointed out by neonatologists.

**Figure 3:**
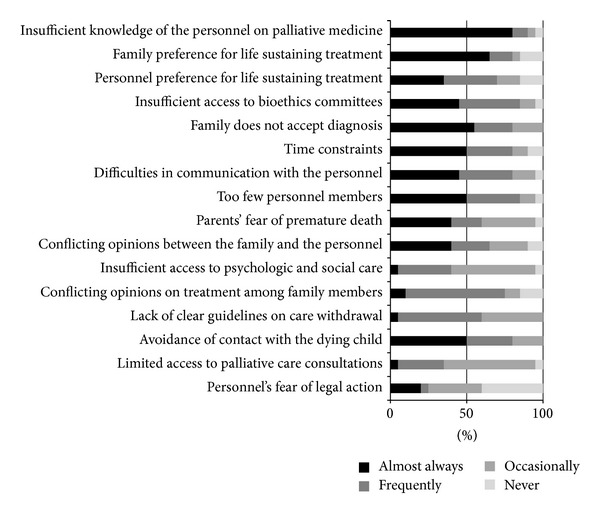
Barriers pointed out by nurses.

The most common barriers pointed out by both groups as occurring almost always were insufficient knowledge of the personnel on palliative medicine and family preference for life sustaining treatment. Detailed results have been presented in Figures 2 and 3. 

There were no differences in responses between the studied groups in regard to their social and demographic data.

## 5. Discussion

Reports on palliative care in perinatology usually concern hospital setting, in particular NICU [14–18]; however numerous publications stress a need for development of home PPC [19–22]. Very few publications worldwide are devoted to the problem of suggesting home palliative care to a newborn with a lethal defect [18, 22–27]. The analysis of structure of patients treated at HHChLR between 2005 and 2011 revealed (unpublished data) that neonates and infants are a growing group of patients referred to palliative care. In 2005 they constituted 8% of hospice patients and in 2011 as much as 46%. This situation results from improving cooperation between HHChLR and neonatological centers in the region. The results of the presented study confirm this observation—the majority of respondents declared cooperation with HHChLR in the past. Although only an every third doctor or nurse ever participate in trainings in PPC, the majority of respondents when asked about principles of management of a child with unfavorable prenatal diagnosis chose in answers accordance with the idea and principles of PPC. However, the study was directed to a selected group of respondents—heads of departments. It is noteworthy that, according to the respondents, families in which lethal defect in a fetus was diagnosed prenatally should be referred to a palliative consultation during pregnancy, which is in concordance with current recommendations. However, within the 7 years of observation none of the families among 53 directed to HHChLR in the first year of life of their child had had any contact with hospice team during pregnancy, despite the fact that the diagnosis was made prenatally. The cooperation with neonatologists can be described as very good, while cooperation between hospice team and obstetricians and centers of prenatal diagnostics requires an active educational and informative campaign. 

An integral element of PPC is the problem of withholding or withdrawal of life sustaining treatment for newborn infants. Despite numerous guidelines, both international and Polish, this problem is still widely discussed and is a source of ethical dilemma for the personnel, which is confirmed by the results of this study and results of other authors. Dangel et al. [28] analyzed the practice of refraining from resuscitation in Poland in patients of neonatological departments and intensive pediatric care units. Decisions on refraining from resuscitation were made in almost 50% of these departments but most often in intensive pediatric care units and 3rd reference neonatological departments. The decision of refraining from resuscitation in over 50% of departments led to limitation or withholding of certain forms of therapy such as dialysis, operative treatment, catecholamines administration, increasing parameters of ventilation, or intubation. Interesting observations were noted in cases where DNR decision was made in a child not on artificial ventilation. Only in 42% of cases patient's discharge home and home hospice palliative care were taken into account. In departments where this line of management was not implemented the argument was that home hospice care was not available and that doctors were not convinced that such management is proper. Additionally, there were differences within this model of management between intensive care units which routinely cooperate with the home hospice team for children and neonatological units which cooperate only occasionally. In the group of neonatological departments children were referred to hospice care more often from 3rd level reference units and least frequently from the 1st level of reference units. The effect of availability of palliative counseling on way of management was also studied by Pierucci et al. [29] who hypothesized that palliative care consultations performed had an impact on the infants' end-of-life care. The results of the current study confirmed this hypothesis; in consulted infants aggressive life sustaining treatment was implemented rarely, duration of treatment in an intensive care unit was shorter, and the families had more frequent referrals for chaplains and social services than families that did not have palliative care consultations.

The Polish law accepts abortion before the fetus is capable of surviving outside the uterus in 3 situations: (1) detection of a serious defect in the fetus, (2) if mother's life is threatened, or (3) pregnancy is an effect of crime. The presented results and opinions on legalization of euthanasia on certain conditions may surprise in the context of religion declared by respondents. More than 95% declared catholic religion, and the Catholic Church is strictly against abortion. However, the respondents only declared their religion in the questionnaire, and not if they are practicing believers, in the view of the so-called conscience clause, acceptable in the Polish law, granting the doctor right to refuse performing a procedure against his philosophy of life. It seems that some of respondents accept an autonomic decision of the mother and her right to abortion in the above-mentioned situations but would not perform the procedure themselves.

In Poland cooperation between hospices and neonatological care centers has improved within the last years and children are directed to home hospice care however implementation of palliative management in neonatological centers still remains controversial and requires further studies. There are reports in the foreign literature on barriers to implementation of palliative care in pediatrics and neonatology in hospital setting; however, in the Polish literature there have been no papers dealing with this subject. In available reports the following problems with implementation of palliative care are listed most frequently: expectations of the parents, rejecting the possibility of an incurable disease in their child, and technological imperative and insufficient knowledge and experience of the personnel. In the presented study the majority of respondents indicated two barriers as occurring “almost always”: insufficient knowledge of the personnel on palliative medicine and family preference for life sustaining treatment. The authors are not surprised that the respondents describe the barrier of inadequate knowledge and experience in palliative care. This barrier undoubtedly results from the fact that palliative care of children is not included or included only marginally in the curriculum of both pre- and postgraduate training. The issue of palliative care has been part of curriculum for medical students in many countries, including Poland, for many years now. However, the Polish programme includes almost exclusively issues of palliative care in adults. Postgraduate training in neonatology does not cover clinical issues of PPC. The only opportunity to gain knowledge is courses and conferences organized by the hospices. This need for training within PPC is confirmed by observations of other authors. The second barrier identified during the study was family preference for life sustaining treatment, and in addition over 60% of doctors and 80% of nurses indicated the barrier “family does not accept diagnosis” as occurring almost always and frequently. These barriers are undoubtedly connected with the problem of appropriate psychological support. The results of the study indicate that only in 45% of approached departments there is a possibility of using psychologic help. Psychological support, as an element of PPC, has been widely discussed in the literature, including support for bereaved parents. From psychological perspective a diagnosis of a lethal defect is a very disruptive life experience. That difficult situation, which touches in a different way the mother, both parents, and the whole family system, requires specialized multidisciplinary care, which respects the perspective of the parents, their emotional and spiritual needs [30–32]. 

The main limitation of the presented study is relatively low number of both groups of respondents. On the other hand, the analysis covers 78% of the whole addressed group and undoubtedly is representative for the Lodz area, where home PPC is covered by the authors of this study. From this point of view, the results are valuable and allow development of future actions which aimed at improving of availability and quality of PPC, both in home-based model and also in neonatological department setting. Narrowing the study group to the neonatological units' staff with omitting obstetricians, midwifes, or geneticists is the next limitation of this study. This first Polish study directly concerning barriers to palliative care may serve as a starting point for future studies.

## 6. Conclusions

Cooperation between home palliative care team and neonatological departments in the studied region may be described as good, and the majority of respondents are aware of the general management principles, according to the idea of PPC, of a child with unfavorable prenatal diagnosis. The most common barriers to introduction of PPC elements in hospital setting identified by neonatologists and nurses are insufficient knowledge of the personnel on palliative medicine and family preference for life sustaining treatment. Medical universities should include elements of PPC in their curricula with special stress on psychological support and communication with the family of the sick child. Understanding attitudes of medical personnel towards PPC and identification of barriers which impair implementation of PPC are a starting point for future efforts to improve the system of care for the youngest patients with life-threatening conditions.

## Figures and Tables

**Table 1:** Social and demographic characteristics of respondents.

	Neonatologists		Nurses	
	*n* = 22		*n* = 20	
Age in years (mean ± SD)	52.64 ± 6.24		46.90 ± 6.60	

	*n*	%	*n*	%

Gender				
Female	14	63.64	20	100.00
Male	8	36.36	0	0
Unit reference level				
1st level	11	50.00	12	60.00
2nd level	9	40.91	6	30.00
3rd level	2	9.09	2	10.00
Religion				
Catholic	19	86.36	18	90.00
Protestant	0	0	0	0
Orthodox	1	4.55	0	0
Islam	0	0	0	0
Atheist	1	4.55	2	10.00
Others	1	4.55	0	0

**Table 2:** Detailed results concerning opinions on withholding resuscitation.

Questions	Answers' options	Number and percentage of respondents who indicated each answer option			
		Neonatologists		Nurses	
		*n*	%	*n*	%
Do you think that a child should be treated till the end, “never give up,” regardless of prognosis and burden of therapy?	Yes, always	2	9.09	3	15.00
	Yes, always when required by parents	4	18.18	2	10.00
	Generally yes, but in a situation described by me and team as hopeless I would suggest palliative care	6	27.27	9	45.00
	No, but I would always consider individual medical and family situation of the child	10	45.45	6	30.00

Which statement best reflects your current opinion?	There are no situations in which resuscitation may be abandoned; we should always fight for a child's life till the end	4	18.18	6	30.00
	If there is no hope that the child may be cured, resuscitation should be initiated because of and on request of the parents	4	18.18	2	10.00
	If there is no hope that the child may be cured, resuscitation should be initiated in order to avoid possible legal claims from family	0	0.00	2	10.00
	Very seldom it may happen that resuscitation prolongs the child's and family's suffering	0	0.00	1	5.00
	Usually if a child is in a terminal stage of a disease, resuscitation prolongs the child's and family's suffering	8	36.36	6	30.00
	If there is no hope that the child may be cured, despite high emotional burden, resuscitation should not be initiated	6	27.27	3	15.00
